# Supplementary material for: Soluble Immune Factor Profiles in Blood and CSF Associated with LRRK2 Mutations and Parkinson’s Disease
Source: bioRxiv. 2025 Mar 24:2025.03.20.644460. Preprint. [Version 1] doi: 10.1101/2025.03.20.644460 (PMC11974741; doi:10.1101/2025.03.20.644460)
Supplement: Supplement 1 [file NIHPP2025.03.20.644460v1-supplement-1.pdf]

bioRxiv preprint doi: https://doi.org/10.1101/2025.03.20.644460; this version posted March 24, 2025. The copyright holder for this preprint (which was not certified by peer review) is the author/funder, who has granted bioRxiv a license to display the preprint in perpetuity. It is made available under aCC-BY 4.0 International license.

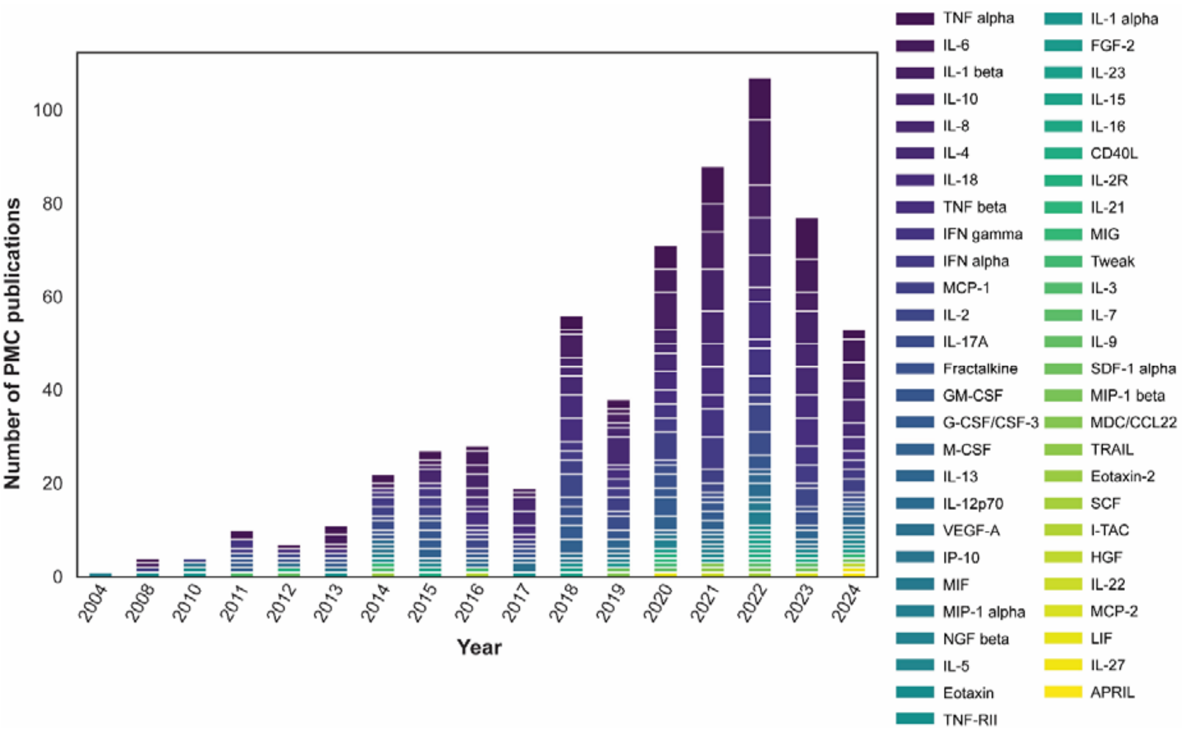

Supplemental Figure 1. The number of PubMed Central (PMC) publications discussing cytokines, *LRRK2*, and Parkinson's disease by publication year. A total of 401 unique papers were retrieved from PubMed and analyzed for 53 cytokines based on keyword search.

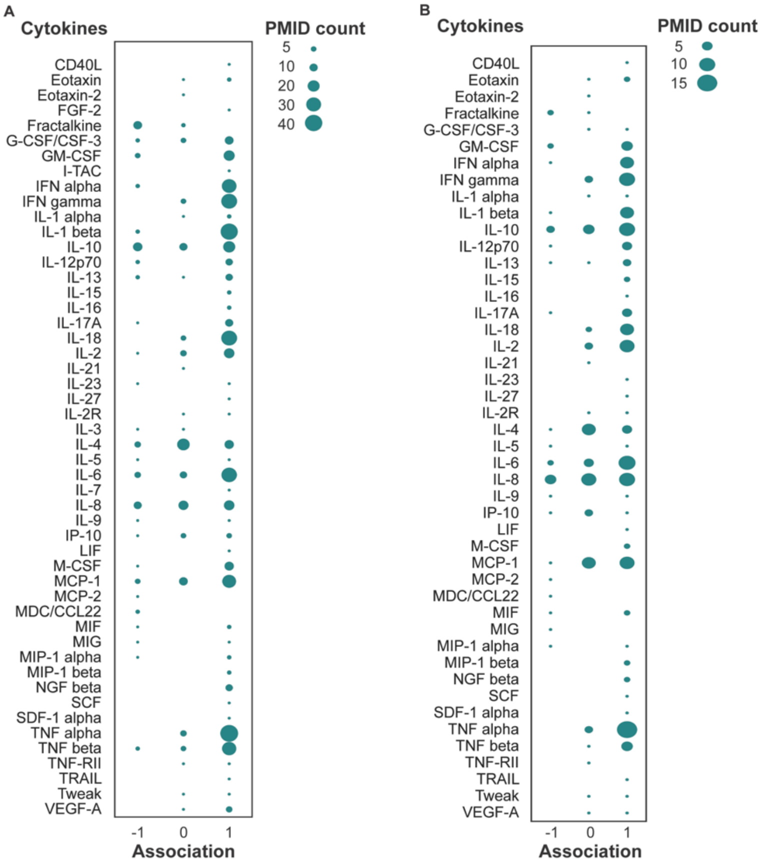

bioRxiv preprint doi: https://doi.org/10.1101/2025.03.20.644460; this version posted March 24, 2025. The copyright holder for this preprint (which was not certified by peer review) is the author/funder, who has granted bioRxiv a license to display the preprint in perpetuity. It is made available under aCC-BY 4.0 International license.

Supplemental Figure 2. GPT-extracted primary literature overview of cytokines and PD. Scores of 1 were given to positive associations between higher cytokine concentrations and PD, -1 to negative associations between lower cytokine concentrations and PD, and 0 to no associations. Bubble size represents the number of papers supporting the association scores. A: Studies across various hosts, such as humans, animals, and cell lines B: Human studies only. Reviews, editorials, letters, and preprints were excluded, totaling 323 papers (out of 401) that were defined as primary literature used in this figure.
